# Production of Ibuprofen Pellets Containing High Amount of Rate Retarding Eudragit RL Using PEG400 and Investigation of Their Physicomechanical Properties

**Published:** 2011

**Authors:** Fatemeh Sadeghi, Hasan Hijazi, Hadi Afrasiabi Garekani

**Affiliations:** 1 Pharmaceutical Research Center and School of Pharmacy, Mashhad University of Medical Sciences, Mashhad, Iran; 2 School of Pharmacy, Mashhad University of Medical Sciences, Mashhad, Iran

**Keywords:** Eudragit RL, Extrusion-spheronization, Ibuprofen, Microcrystalline cellulose, Pellets, PEG400

## Abstract

**Objective(s):**

The aim of this study was to investigate the possibility of production of ibuprofen pellets with high amount of rate retarding polymer by aid of PEG400 as plasticizer.

**Materials and Methods:**

Polyethylene glycol (PEG400) in concentrations of 1, 3 or 5% w/w with respect to Eudragit RL was used in production of pellets containing 60% ibuprofen and 40% excipient (2% polyvinylpyrrolidone (PVP), 7.6 or 0% microcrystalline cellulose (MCC) and 30.4 or 38% Eudragit RL). Physicomechanical and release properties of pellets were evaluated.

**Results:**

In presence of PEG400, formulations containing 30.4% Eudragit RL and 7.6% MCC could easily form pellets. In formulations without any MCC pellets were obtained only in presence of 3 or 5% PEG400. Pellets containing MCC with 0 or 1% PEG400 showed brittle properties but those with 3% or 5% PEG400 showed plastic nature under pressure. Elastic modulus dramatically decreased with increasing PEG400 indicating softening of pellets. This was due to shift of Eudragit structure from glassy to rubbery state which was supported by DSC studies. Mean dissolution time (MDT) increased with addition of 1 or 3% PEG400 but this was not the case for pellets with 5% PEG400.

**Conclusion:**

Overall PEG400 is a potential plasticizer in production of pellets based on Eudragit RL and ibuprofen. The ease in process of extrusion-spheronization, increasing the mean dissolution time and change in mechanical properties of pellets from brittle to plastic behavior were advantages of using PEG400. Changes in mechanical properties of pellets are important when pellets are intended to be compressed as tablets.

## Introduction

Multiple unit sustained release dosage forms comprising granules, microcapsules, pellets or spheroids are the most popular oral sustained release dosage forms due to their several advantages (1, 2).

Preparation of sustained release matrix pellets using rate retarding polymers are considered important because they have all the benefits of multiple unit systems and are produced in single step process without any need for further coating procedure. Extrusion spheronization is one of the widely used methods for production of pellets especially when the dose of drug is high. One of the major limitations in preparation of sustained release pellets with high drug loading using extrusion spheronization technique is the necessity for the use of a pelletization aid such as microcrystalline cellulose (MCC) in order to provide plasticity and proper cohesive properties for the wet mass. This would limit the use of sustained release polymers in formulation of pellets. Therefore pellets produced on the basis of this method usually need polymeric coating in order to retard drug release rate. Recently the use of release retarding materials along with MCC for production of sustained release matrix pellets has been noticed. Eudragit RL, Eudragit RS (3) and chitosan (4) are among polymers used for this purpose. 

Furthermore compaction of multiparticulates into tablets is becoming more popular. Pellets which are intended to be compressed into tablets should deform under applied load without fracture. Alterations in mechanical properties of either coated or uncoated pellets from brittle to plastic nature makes them a suitable substrate for compression in the form of tablets as these changes could prevent cracking of pellets and/or their coating under the compression force and therefore limit the changes in the release properties after compression. Abbaspour et al showed that curing (thermal treating) of Eudragit based pellets containing 40 or 60% ibuprofen could bring about some changes in mechanical properties of these pellets and change their behavior from brittle to plastic under the mechanical test (5).

Plasticizers are widely used in film coating and the production of soft gelatin capsules. In general, incorporation of a plasticizer increases plasticity and changes the flexibility, tensile strength and adhesion properties of polymers (6). It has been shown that inclusion of plasticizer into matrices or pellets could profoundly change their mechanical and release properties (7, 8). 

As stated before in production of pellet by extrusion-spheronization MCC provide proper plasticity in wet mass and facilitate the process of extrusion and spheronization. In a study by Abbaspour et al it was shown that Eudragit based ibuprofen pellets with 60% drug loading could easily be obtained in presence of at least 10% MCC in their formulations. The aim of this study was to prepare ibuprofen pellets with high loading of drug (60%) and to replace the MCC with rate controlling polymer of Eudragit RL as much as possible with aid of PEG400 as plasticizer and to investigate the physicomechanical and release properties of pellets. The rational of using PEG400 was based on this concept that plasticizer (PEG400) could possibly provide the proper plasticity for the wet mass with less or no MCC and therefore replacement of MCC with rate retarding polymer would be probable.

## Material and Methods

***Material***

Ibuprofen and microcrystalline cellulose (Avicel PH101) were provided by Darupakhsh (Tehran, Iran), Eudragit RL PO was gift from Rohm Pharma GmbH (Darmstadt, Germany), polyvinylpyrrolidone (PVP K30) was supplied by Fluka (Switzerland), PEG400, sodium hydroxide and dihydrogen potassium phosphate were supplied by Merck (Germany).

***Methods***

***Preparation of pellets***

The composition of different formulations of pellets containing 60% ibuprofen and 40% excipient has been shown in  Table 1. The plasticizer concentration on pellet formulation 

**Table 1.** Ingredients used for production of pellets containing 60% ibuprofen and 40% excipients along with PEG400 as plasticizer.

Ingredient	Formulations							
	F1	F2	F3	F4	F5	F6	F7	F8
Ibuprofen (%w/w)	60	60	60	60	60	60	60	60
MCC (%w/w)	7.6	7.6	7.6	7.6	0	0	0	0
Eudragit RL (%w/w)	30.4	30.4	30.4	30.4	38	38	38	38
PVP K30 (%w/w)	2	2	2	2	2	2	2	2
PEG400 (%w/w with respect to Eudragit RL)	0	1	3	5	0	1	3	5
Water (%w/w with respect to total weight of wet mass)	35.9	34.2	33.3	33.3	35.9	34.2	32.4	29.5

was 1, 3 or 5% w/w based on the weight of Eudragit RL. To prepare pellets, the solid ingredients of each formulation (50 g) were mixed using a kitchen mixer for 10 min. The required amount of water was slowly added to the dry blend to make a proper wet mass. For those pellets containing plasticizer, the plasticizer was mixed with half of the amount of water required for preparation of pellets and added to the powder mixture. Then proper wet mass was obtained with addition of further amount of water. The wet mass was passed through a screw extruder () fitted with a 1 mm screen at 120 rpm. The extrudates were processed in a spheronizer () fitted with a cross-hatched plate rotated at 1000 rpm for 2 min. The obtained pellets were dried at 40 C for 10 hr in a conventional hot air oven.

***Mechanical tests***

The crushing strength (the load needed to break the pellets) or yield point (the load needed to begin plastic deformation) of 10 pellets in the size range of 0.85-1.00 mm was determined using Material Testing Machine (Hounsfield, England). The speed of the upper mobile platen fitted with a 1 kN load cell was set at 1mm/min. Elastic modulus and force-displacement graphs were obtained by a computer system attached to the apparatus (QMAT, Hounsfield, England). 

***Dissolution studies***

The dissolution tests were carried out on accurately weighed samples (n= 6) containing 300mg of ibuprofen in automated dissolution testing equipment (Pharma test, Germany) using USP apparatus I, at 100 rpm, in medium of 900 ml phosphate buffer solution of pH 7.2, at 37 °C. The samples were taken from the vessels by a peristaltic pump (Alitea, Sweden), and assayed at 265 nm by a multi-cell transport spectrophotometer (Shimadzu, Japan). Two distinct absorbance peaks in the UV range could be observed for ibuprofen; a high peak at 221 nm and the shorter one at 265 nm. As dilution of samples during automated dissolution test was impossible, the shorter peak at 265 nm was chosen for determination of ibuprofen based on Costa et al. (9). 

Model independent approach was used to compare the dissolution data. For this purpose mean dissolution time (MDT) was calculated for each formulation by following equation (9):

MDT = Σ t_i_ˉ.ΔM_i_ / Σ ΔM_i_ (1) 

t_i_ˉ = (t_i_ + t_i+1_) / 2 (2) 

ΔM_i_ = (M_i+1_^_^ M_i_) (3)

Where tˉ_i_ is the midpoint of the time period during which the fraction ΔM_i_ of the drug has been released from the dosage form. A high MDT value for a drug delivery system means that it has a slow in-vitro drug release.

***Scanning electron microscopy (SEM)***

The surfaces of pellets were morphologically characterized using SEM. The samples were mounted on Al stub, sputter-coated with a thin layer of Pt using sputter coater (Polaron, England) under Argon atmosphere, and then examined using SEM (LEO1450VP, England).

***Determination of size and size distribution of pellets***

The pellets were sieved using nest of standard sieves (1180, 1000, 850 and 710 μm) shaken for 5 min on a sieve shaker (Retsch-Germany). The weight of each fraction was determined and the cumulative frequency of undersize on probability scale was plotted against log of size. Mean particle size and geometric standard deviation were determined from the plot.

***Differential scanning calorimetery (DSC)***

DSC analysis was performed on Eudragit RL, ibuprofen and grounded pellets containing 0 and 3% PEG using a differential scanning calorimeter (Mettler Toledo DSC 822e, Switzerland) and STARe software version 7.01 (Mettler Toledo, Switzerland). The instrument was calibrated with an indium standard. Samples (7–10 mg) were weighed and sealed into aluminum pans. The DSC runs were conducted over a temperature range of 25–100 ºC at a rate of 5 ºC/min. All tests were run under a nitrogen atmosphere.

***Statistical analysis***

One way analysis of variances was used for statistical comparison of mechanical and dissolution test results.

## Results

The results of mean particle diameter are presented in Table 2. The pellet mean diameter increased slightly with addition of plasticizer to the formulations. However increase in concentration of plasticizer did not affect the particle size of the pellets.

**Figure 1.**
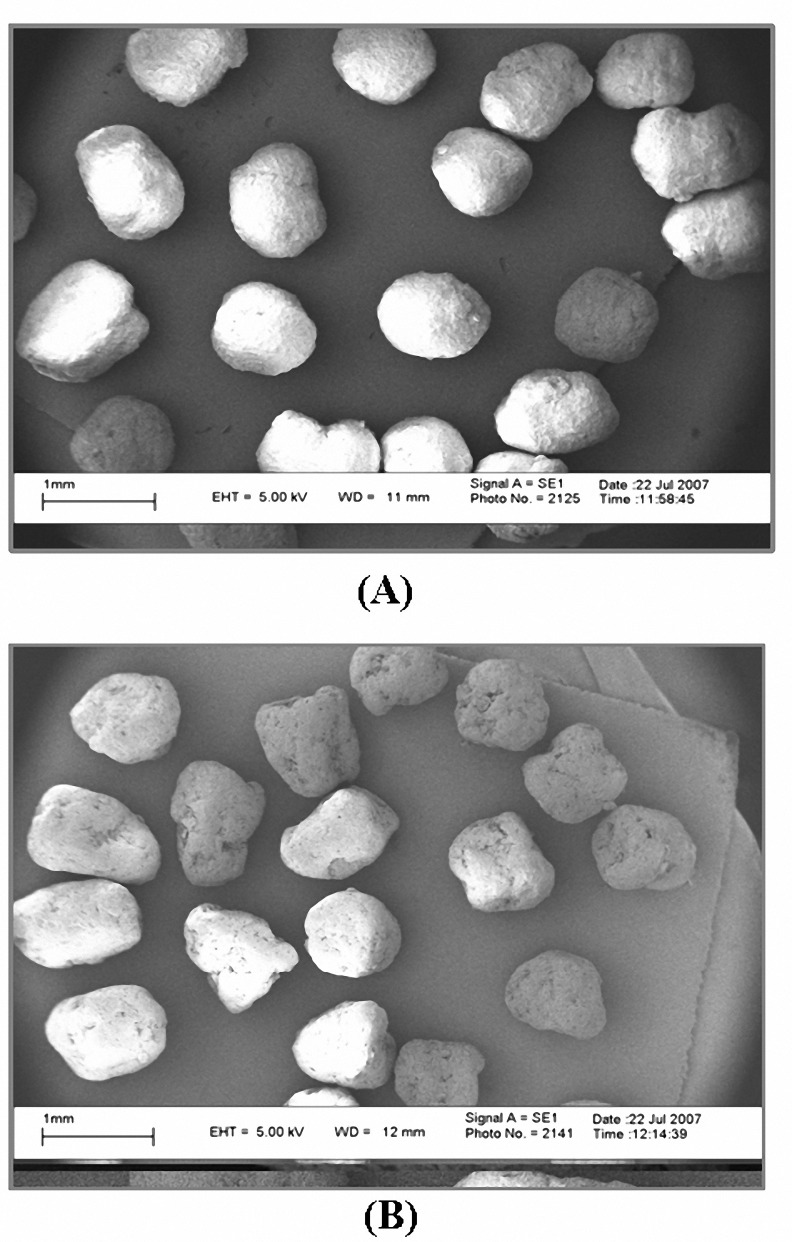
Scanning electron micrograph of pellets (A) F3 formualtion and (B) F7 formulation.

Figure 1 show the scanning electron micrograph for formulation F3 and F7. Pellets prepared from formulations containing MCC (F3) were nearly spherical. However the shape of pellets prepared from formulations without MCC (F7) showed some deviation from sphericity. 

**Table 2.** Geometric mean particle diameter and geometric standard deviation for pellets obtained from different formulations.

Formulation	d_g_ (µm)	_g_
F1	825	1.3
F2	935	1.4
F3	923	1.2
F4	927	1.1
F5	No pellets were obtained	-
F6	No pellets were obtained	-
F7	893	1.2
F8	920	1.3

The results of mechanical test of the pellets are shown in Table 3. Pellets containing MCC with 0 or 1% plasticizer showed brittle behavior under the mechanical test and addition of 1% plasticizer led to decrease in crushing strength and elastic modulus of the pellets (*P*< 0.05). However formulations of F3 and F4 with 3 or 5% plasticizer showed plastic deformation under the mechanical test and the yield point decreased with increase in plasticizer concentration. Formulations F7 and F8 with no MCC and containing 3 or 5% plasticizer also deformed plastically under the mechanical test.

**Figure 2.**
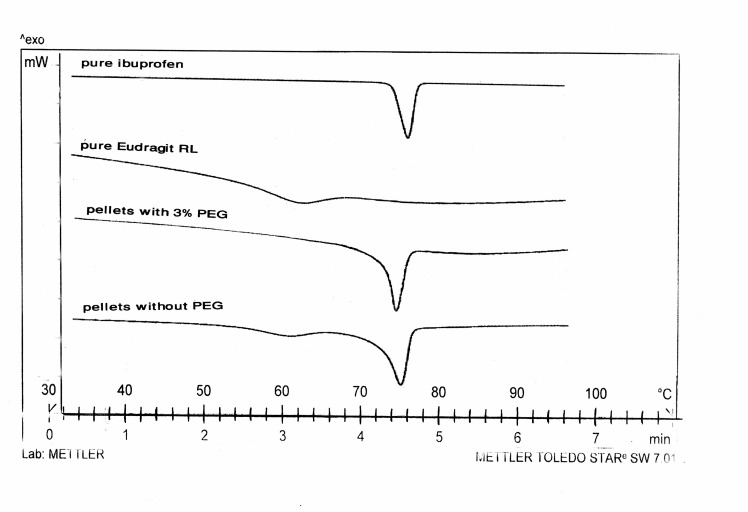
DSC scans of pure Eudragit RL, pellets without PEG, pellets with 3% PEG and ibuprofen.

The thermograms for pure Eudragit RL and pellets of formulation F1 (without PEG) and formulation F7 (with 3% PEG) are depicted in Figure 2. The onset of the peak for glass transition temperature of pure Eudragit RL appeared at about 55 ºC and for melting of ibuprofen at 76 C.

**Table 3.** he results of mechanical test of pellets.

Formulation	Crushing strength (N)	Yield point (N)	Elastic modulus (Mpa)
F1	3.2±0.20	-	105±5
F2	2.4±0.15	-	62±4.1
F3	-	1.8±0.08	35±1.9
F4	-	1.6±0.13	29±2.0
F5	No pellets were obtained	-	-
F6	No pellets were obtained	-	-
F7	-	1.5±0.10	22±1.8
F8	-	1.3±0.11	18±1.1

**Figure 3.**
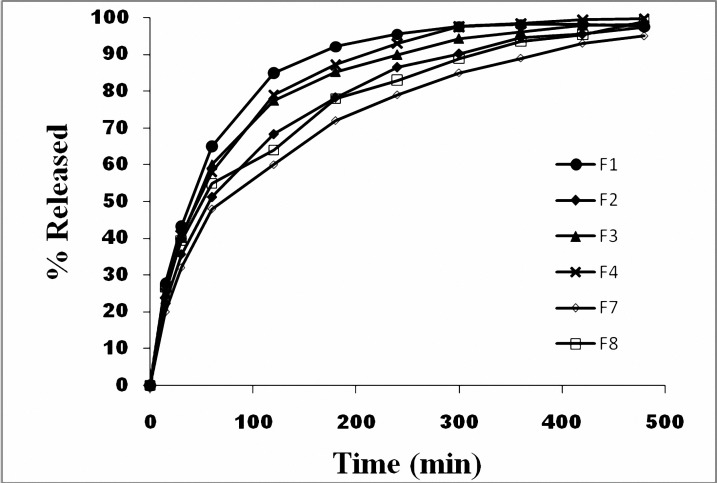
Dissolution profiles of ibuprofen pellets.

The dissolution profiles of the pellets are shown in  Figure 3. The results of mean dissolution time calculated from release profiles (Table 4) showed that for formulations containing MCC, increase in concentration of plasticizer up to 3% increased MDT of the pellets significantly (*P*< 0.05). However there were no significant differences between MDT of formulation F4 (containing 5% plasticizer) with F1 (*P*> 0.05). Formulation F7 containing 3% PEG400 and 38% Eudragit RL, showed the highest MDT among different formulations.

**Table 4.** MDT calculated for different formulation.

Formulation	MDT (min)
	Uncured pellet
F1	51±2.5
F2	77.8±4.5
F3	68.5±5.5
F4	53.5±3.4
F5	No pellets were obtained
F6	No pellets were obtained
F7	82.3±4.1
F8	76.3±5.3

## Discussion

MCC due to its unique properties has been the excipient of choice for pellet production using extrusion spheronization technique. This material could facilitate the extrusion process, improve plasticity of the wetted mass and enhance spheronization (10). MCC unique properties include high surface area and high porosity which give it the ability of absorbing and retaining high quantity of water and also providing the proper rheological properties to wetted mass. In recent studies attempts have been made to use rate retarding polymers Eudragit RL or RS along with MCC in process of extrusion spheronization in order to achieve sustained release of drug from pellets with different drug loadings (3). In present study pellet containing 60% ibuprofen and 40% excipient was prepared aiming to replace the MCC with rate controlling polymer of Eudragit RL as much as possible and investigate the effect of addition of PEG400, as a plasticizer, in this regard.

Addition of PEG400 was found to be suitable for preparation of pellets containing Eudragit RL. Formulation F1 (with 7.6% MCC and no plasticizer) was unable to form proper extrudate after one run through the extruder and therefore to obtain acceptable extrudate this formulation was passed one more time through the extruder. Formulation F5 (with no MCC and no plasticizer) could not be processed in the extruder at all and therefore no pellets could be obtained from this formulation. Addition of 1% w/w of plasticizer based on Eudragit RL weight, in formulation containing 7.6% MCC and 30.4% Eudragit RL, facilitated the process of extrusion for formulation F2 and proper extrudate was easily obtained after just one passage through the extruder. Increase in concentration of plasticizer in formulations F3 and F4 also gave the same results. Pellets obtained from formulations of F2 to F4 were nearly spherical. Figure 1 shows the scanning electron micrographs of pellets obtained from F2 Formulation.

Addition of 1% plasticizer to formulation containing 38% Eudragit RL and no MCC (formulation 6) could not lead to preparation of proper extrudate and therefore no pellets were obtained from this formulation. But following addition of 3% or 5% PEG400 (formulations F7 and F8) the wet mass could be processed through the extruder and form pellets. However the extrusion of the wet mass was not performed as easy as that for formulations of F3 or F4. Furthermore the shape of the pellets obtained showed slight deviation from spherecity (Figure 1).

The required amount of water for preparation of wet mass was different for various formulations. It has been shown that the amount of water needed to prepare wet mass in process of extrusion spheronization is dependent on the amount and properties of formulation components (11). The amount of water required for preparation of wet mass which is depicted in table 1 decreased by addition of plasticizer in formulation. This was attributed to the liquid nature of plasticizer and its interaction with Eudragit RL which led to enhanced cohesiveness. Felton et al. and Fujimori et al. showed that PEG400 is a suitable plasticizer for acrylic resin polymers (12, 13).

The results of sieve analysis showed that the percent of fine particles and agglomerates were very low indicating the proper moisture content of wet mass. Fielden et al reported that less moisture content of wet mass could lead to formation of fine particles in process of spheronization and high moisture content could result in agglomeration (14). Addition of PEG400 resulted in preparation of pellet with larger mean diameter (Table 2). Increase in cohesive nature and plasticity of wet mass in presence of plasticizer could account for increase in diameter of the pellets as increased plasticity of wet mass could prevent breaking of extrudates into small pieces during spheronization.

The results for mechanical test of pellets (Tables 3) indicate that pellets with 0 or 1% plasticizer (formulation F1 and F2) showed brittle behavior under the load and therefore the values of crushing strength have been reported for them. Similarly Abbaspour et al reported that ibuprofen Eudragit based pellets with 60% drug loading showed brittle properties under the mechanical test (3). Pellets with 3 or 5% PEG (formulation F3 and F4) showed plastic deformation nature and have not been fractured under the load and therefore the yield point has been reported for these pellets. Overall the addition of PEG400 decreased the elastic modulus of the pellets significantly (Table 3) (*P*< 0.05). Similarly Wang et al. reported that addition of plasticizer reduced the crushing strength and elastic modulus of MCC pellets prepared by addition of aqueous dispersion of Eudragit RS (Eudragit RS 30D) as granulating liquid (7). 

Pellets with no MCC and containing 3% or 5% plasticizer (formulation F7 and F8) also showed plastic properties under the load and exhibited the lowest yield points and elastic modulus, indicating that these pellets were softer than those containing MCC and same amount of plasticizer. The transition of pellet behavior from brittle to plastic nature in presence of PEG400 was due to softening of polymer and shift of Eudragit structure from glassy to rubbery state which was supported by DSC studies (Figure 2). The transition peak for Eudragit RL and the peak related to the melting of ibuprofen are clearly visible in pellets of formulation F1 (without PEG400). However no peak for the transition of Eudragit RL could be observed in thermogram of pellets of formulation F7 (with 3% PEG400), indicating the rubbery or plastic state of polymer in presence of PEG400, at ambient temperature. 

The results for MDT (table 4) showed that addition of 1 or 3% PEG400 to pellets containing Eudragit RL and MCC (formulations F2 and F3) increased the MDT compared to those pellets with no plasticizer. However presence of 5% plasticizer (Formulation F4) did not affect the MDT for these pellets significantly (*P*> 0.05). In pellets with no MCC and higher amounts of Eudragit RL (formulation F7 and F8) the MDT has been increased significantly compared to formulations with MCC and no plasticizer. This is attributed to increase in the amount of retarding polymer in formulation. Sadeghi et al. on their research on solid dispersion systems of Eudragit RS (15) and ethylcellulose (16) found that addition of plasticizer decreased the release rate of drug from tablets prepared of solid dispersions systems. Zhu et al. also showed that addition of trietyl citrate into the direct compressed tablets of chlorpheniramine maleate and Eudragit RS led to formation of more homogenous matrix and therefore lower drug release rate (8). Formation of more homogeneous Eudragit RL matrix following the addition of 1 or 3% plasticizer could also explain the observed results in this study. According to Wang et al. a plasticizer may function as an adhesion promoter (7) and therefore could help to form more homogenous matrix in pellet structure. 

However as PEG400 is a water soluble plasticizer, therefore when used in higher concentrations (5%) it could provide more pores for drug release following its dissolution and therefore the polymeric matrix would be more porous for pellets containing 5% plasticizer. This would explain the lower MDT for the pellets prepared from formulations containing 5% plasticizer compared to those with 3% plasticizer.

## Conclusions

The results of this study revealed that addition of proper plasticizer may provide the possibility of replacement of MCC with release retarding polymer such as Eudragit RL in process of extrusion-spheronization. However this replacement could not lead to desired sustained release for pellets containing high load of drug and therefore the coating process still may be required to retard the release of drug. The ease in pellets production process and changes in mechanical properties of pellets would be the advantages of using plasticizer in production of pellets containing Eudragit RL in their formulation. The changes in mechanical properties of pellets are beneficial especially when the compaction of the pellets as tablets is desired. The concentration of plasticizer has a great influence on mechanical properties of the pellets. As pellets with 5% plasticizer did not show any additional advantages over those containing 3% plasticizer it was concluded that the lower concentration of plasticizer i.e. 3% would be more appropriate in formulation of pellets with Eudragit RL in their structure.
